# Atlanta Medical College

**Published:** 1866-08

**Authors:** 


### EDITORIAL AND MISCELLANEOUS.

### ATLANTA MEDICAL COLLEGE.

This institution is now in a condition to afford abundant facilities for the study of all the branches of the "healing art." The building, during the scourge of war, was so damaged that an outlay of several thousand dollars became necessary to place it in a condition suitable to the purposes for which it was constructed. This has been done, and now, in point of appearance, it is equal to that of any time since it was built. In fact, the damage to the house, apparatus, museum and library has been repaired, and they are complete in all respects, except the library and museum, which still exhibit evidences of the despoiler's hand. A little more time and nothing will be wanting in the institution to remove the last mark of war's destructive hand.

The amount necessary for these purposes has been donated by the City Council of Atlanta, and while we feel grateful for this timely beneficence, we are encouraged to still greater efforts in making the institution an honor to the City, and a blessing to mankind.

The class of the present session, is respectable in size—even larger than the most sanguine would expect in the ruined condition of the country. In point of devotion to study and gentlemanly deportment, no institution was ever more signally favored. It is by such alumni that the respectability of Medical Institutions is sustained, and the honor and usefulness of the medical profession preserved.

The exhibition of such earnest efforts to advance in the study of science, excites the teachers to increased efforts, in their course of instruction, to give every facility for rapid and permanent improvement.

The present Session certainly affords a more perfect and thorough course of teaching than has been offered by this institution to any class previously. Besides the addition of one chair to the curriculum of studies, a much more perfect system of Clinical instruction has been adopted. The daily examination of, and prescribing for, fifteen or twenty patients in presence of the class, affords an amount of practical study entirely sufficient for the medical student. That this course of study may be still more practical and useful, the opportunity is afforded, to such as desire it, of assisting in examinations, writing and putting up prescriptions, &c. In addition to this, the regular Medical and Surgical Clinique, occupy each, one hour, two days in the week. These hours are occupied with lectures on interesting medical and surgical cases.

